# Clinical significance and biological function of interferon regulatory factor 1 in non-small cell lung cancer

**DOI:** 10.3389/fphar.2024.1413699

**Published:** 2024-06-10

**Authors:** Jialin Su, Shuhua Tan, Yuning Li, Xinglong Chen, Jiasi Liu, Yongzhong Luo, Changqie Pan, Lemeng Zhang

**Affiliations:** ^1^ Thoracic Medicine Department, Hunan Cancer Hospital, Changsha, Hunan Province, China; ^2^ School of Life and Health Sciences, Hunan University of Science and Technology, Xiangtan, Hunan Province, China

**Keywords:** interferon regulatory Factor-1, non-small cell lung cancer, interleukin-2, chemoimmunotherapy, inflammatory pathway

## Abstract

The clinical application and biological function of interferon regulatory factor 1 (IRF1) in non-small cell lung cancer (NSCLC) patients undergoing chemoimmunotherapy remain elusive. The aim of this study was to investigate the predictive and prognostic significance of IRF1 in NSCLC patients. We employed the cBioPortal database to predict frequency changes in IRF1 and explore its target genes. Bioinformatic methods were utilized to analyze the relationship between IRF1 and immune regulatory factors. Retrospective analysis of clinical samples was conducted to assess the predictive and prognostic value of IRF1 in chemoimmunotherapy. Additionally, A549 cells with varying IRF1 expression levels were constructed to investigate its effects on NSCLC cells, while animal experiments were performed to study the role of IRF1 *in vivo*. Our findings revealed that the primary mutation of IRF1 is deep deletion and it exhibits a close association with immune regulatory factors. KRAS and TP53 are among the target genes of IRF1, with interferon and IL-2 being the predominantly affected pathways. Clinically, IRF1 levels significantly correlate with the efficacy of chemoimmunotherapy. Patients with high IRF1 levels exhibited a median progression-free survival (mPFS) of 9.5 months, whereas those with low IRF1 levels had a shorter mPFS of 5.8 months. IRF1 levels positively correlate with PD-L1 distribution and circulating IL-2 levels. IL-2 enhances the biological function of IRF1 and recapitulates its role *in vivo* in the knockdown group. Therefore, IRF1 may possess predictive and prognostic value for chemoimmunotherapy in NSCLC patients through the regulation of the IL-2 inflammatory pathway.

## Introduction

Lung cancer remains the leading cause of cancer-related deaths worldwide, with NSCLC accounting for 80%–85% of these cases. Recently, the combination of immune checkpoint inhibitors (ICIs) and chemotherapy has received a lot of attention, and this combination is recommended as an initial treatment option (Lantuejoul et al., 2020). However, the discovery of prognostic and predictive biomarkers for chemoimmunotherapy holds significant clinical importance. IRF1 is the earliest interferon regulatory factor, and its expression regulates the malignant biological behavior of tumor cells (Kirchhoff et al., 1993), IRF1 inactivation increases the risk of tumorigenesis (Nozawa et al., 1999). Previously, we report that IRF1 increases chemotherapy sensitivity in NSCLC by modulating apoptosis and autophagy (Zhang et al., 2022). However, the clinical significance of IRF1 in NSCLC patients who receiving both chemotherapy and immunotherapy remains largely unknown.

IRF1 was associated with pro-inflammatory cytokines release, lymphocyte growth and differentiation, innate and acquired immunity, which might be closely related to immunotherapy efficacy (Miyamoto et al., 1988; Feng et al., 2021). Since IRF1 was closely related to efficacy of both immunotherapy and chemotherapy, its clinical significance in chemoimmunotherapy is worth studying. Therefore, we first investigated the clinical significance and biological functional role of IRF1 by bio-informatics methods. Then we analyzed the efficacy of chemoimmunotherapy based on different IRF1 levels by clinical samples retrospectively. The function of IRF1 in proliferation, migration, and invasion were analyzed *in vitro*. The effect of IRF1 on tumorigenic ability of tumor cells was analyzed *in vivo*. A comprehensive, in-depth understanding of the clinical application and biological function of IRF1 in NSCLC chemoimmunotherapy, is of great theoretical and practical significance for NSCLC treatment.

## Materials and methods

### Frequency changes in the IRF1 gene

The mutation status of IRF1 was analyzed using the cBioPortal database (http://www.cbioportal.org/) (Gao et al., 2013). The name of the IRF1 gene was used for the mutation-related analysis and visualization.

### Genomics enrichment analysis (GSEA) of IRF1

Samples were grouped based on the expression levels of the IRF1 gene, and differential expression analysis between groups was performed using the Limma package in R software (Version 3.10.3, http://www.bioconductor.org/packages/2.9/bioc/html/limma.html) (Kerr, 2003). To analyze the pathways enriched by IRF1, GSEA analysis was performed using the R package clusterProfiler with MSigDB v7.2 (http://software.broadinstitute.org/gsea/msigdb/index.jsp) (Shannon et al., 2003) symbols. GMT served as a rich background, and the analysis was performed based on the IRF1 level combined with the sample grouping information.

### Relationship between IRF1 and immune regulatory factors TMB and MSI

TMB is defined as the total number of base mutations per million cells in a tumor that can stimulate the production of tumor-specific and highly immunogenic antibodies, and has been recognized as a novel target for predicting the effectiveness of tumor immunotherapy. MSI is defined as the phenomenon wherein a new micro-satellite allele appears at a micro-satellite site in a tumor compared to normal tissue owing to the insertion or deletion of repetitive units, which leads to functional defects in DNA mismatch repair in the tumor tissue. The expression matrices of IRF1 and immune regulatory gene sets from dataset were extracted, and the Spear-man correlation coefficients between IRF1 and various genes, TMB, and MSI in the immune regulatory gene set were calculated using the cor. test function in R software.

### Prediction of the IRF1 target genes

Target genes of IRF1 were predicted using the TRRUST Database (https://www.grnpedia.org/trrust/) (Han et al., 2018). To ensure that the interactions in the database were experimentally validated, the target genes were selected using MeSH vocabulary queries and continuously improving sentence-based text-mining algorithms, and carefully proofread manually after mining. Protein-protein interaction (PPI) was performed using the STRING (version 11.0, http://www.string-db.org/) database after target gene selection for IRF1 (Szklarczyk et al., 2021). Required Confidence (combined score) > 0.7 was selected as the threshold for PPI, and relevant files were downloaded in tsv format. After obtaining the PPI relationship pair file, Cytoscape software (version 3.4.0, http://chianti.ucsd.edu/cytoscape-3.4.0/) was used to construct the network (Shannon et al., 2003).

### Correlation analysis between IRF1 expression and immunotherapy

The TIDE score was calculated based on the mRNA expression matrix (http://tide.dfci.harvard.edu/) to predict immune therapy response. The difference in the TIDE scores of IRF1 between the high- and low-expression groups was compared using the R package ggpubr (version 0.6.0).

### Clinical sample collection and analysis

Between February 2021 and December 2022, 168 NSCLC patients with receiving first-line chemoimmunotherapy from Hunan Cancer Hospital, China, were analyzed retrospectively. NSCLC was diagnosed by pathological examination. Epidermal growth factor receptor (EGFR) and anaplastic lymphoma kinase (ALK) mutation was were excluded. Information, including age, gender, pathological type, Lung adenocarcinoma (LUAD) and Lung squamous cell carcinoma (LUSC), PD-L1 (%), smoking status, PS score, TNM stage were collected. The TNM classification was according to National Comprehensive Cancer Network Classification Standard Eight Edition. All the patients were received first-line chemotherapy plus ICIs. Treatment regimens for LUSC was nab-paclitaxel 260 mg/m^2^intravenously (IV) plus carboplatin AUC five IV days 1once every 3 weeks plus ICIs. Treatment regimens for LUAD was pemetrexed 500 mg/m2 IV plus carboplatin AUC five IV days 1once every 3 weeks plus ICIs. The measurable tumor was evaluated once every 6 weeks in the first 12 months and once every 9 weeks in year two and beyond using Response Evaluation Criteria in Solid Tumors (RECIST) version1.1. The efficacy of chemoimmunotherapy were classified into complete response (CR), partial response (PR), stable disease (SD), progressive disease (PD). The combination rate of CR and PR were as objective response rate (ORR). Progression-free survival (PFS) was defined as the length of time during and following primary treatment without progression, as demonstrated by radiological and clinical examinations. The study was granted by the Ethics Committee of Hunan Cancer Hospital and conducted in compliance with the principles of the Declaration of Helsinki. Written informed consent was obtained from all the enrolled patients.

### Circulating biomarker analyze

Once whole blood was collected in tubes containing EDTA anticoagulant, samples were centrifuged at 400 *g* for 2 hours at 4°C. The plasma samples have been collected and then stored at −80°C before to utilization. Chemical ELISA Kit (Huamei, Wuhan, China) was used to assess TNFα, interferon γ, IL-2, and IL-6. Hunan Cancer Hospital’s central laboratory examined B cells, natural killer (NK), CD3, CD4, and CD8 percentages, as well as serum tumor indicators.

### Immunohistochemistry (IHC)

Fixed or fresh tumor biopsy samples were obtained from patients before treatment. PD-L1 and IRF1 expression was evaluated by IHC staining as described before (Zhou et al., 2014; Zhang et al., 2022). PD-L1 positivity was defined as TC≧1%.

### IRF1 quantification by IHC

IRF1 staining was scored independently by two pathologists and was calculated using a previously defined scoring system (Zhou et al., 2014). Briefly, the proportion of positive tumor cell was scored as: 0 = <5%; 1+ = 5–20%; 2+ = 21–50%; 3+ = 50–70% and 4+ = 70–100%. The intensity was arbitrarily scored as 0 = weak (no color or light blue), 1 = moderate (light yellow), 2 = strong (yellow brown), and 3 = very strong (brown). The overall score was calculated by multiplying the two scores obtained from each sample. a score of ≥8 was defined as high IRF1 expression and scores of <8 defined low IRF1 expression. All the enrolled patients were divided into two groups according to the expression levels of IRF1: a group with high IRF1 levels and a group with low IRF1 levels (Supplementary Figure S1). The relationship between IRF1 levels and clinical characteristics was subsequently analyzed.

### Reagents and antibodies

IL-2 (sigma)10 ng/mL was used for 12 h. IRF1Ab was from Santa Cruz (Santa Cruz, CA, USA) and PDL1 Ab 22C3 was from Dako Agilent. Goat anti-rabbit secondary antibodies were purchased from Thermo Fisher Scientifc Inc. (Waltham, MA, USA). CCK-8 solution was from Guangzhou Yitao Biotechnology Co, LTD (Guangzhou, China). Nude mice were from Hunan Slack Jingda Experimental Animal Co., LTD. (Hunan, China). Matrigel was purchased from Corning Life Sciences (Wujiang) Co., LTD (Jiangsu, China). Phosphate buffer salt solution (PBS), trypsin, Dulbecco’s modified eagle medium (DMEM), fetal bovine serum (FBS) were purchased from Gibco Life Technologiesin (New York,USA). Crystal violet staining solution, 4% paraformaldehyde fix solution was purchased from Shanggong Bioengineering (Shanghai) Co., LTD (Shanghai, China).

### Cell culture and transfection

The American Type Culture Collection (Manassas, VA, USA) provided the human lung cancer cell lines A549. A titer of 1 × 109TU/mL was generated for IRF1 shRNA and overexpression lentiviral vectors (Hanyin, Shanghai, China). Using 5 μg/mL polyamine in RMI-1640 media, these vectors were transfected into cells at a multiplicity of infection (MOI) of 20:1. The cells were grown in new culture media outlets for 48 h following a 4-h transfection. The effectiveness of IRF1 overexpression (OE) and knockdown (KD) was evaluated using flow cytometry and Western blotting.

### CCK-8 cell proliferation experiment

After cell concentration was adjusted, cells were inoculated into 96-well plates with 5×10^4^ cells per well. Cells in each group were provided with four multiple Wells and blank controls, and cultured in an incubator for 0h, 24 h and 48h, with 10 µL CCK-8 solution per well and incubated for 2 h away from light. optical density (OD) at 450 nm was measured. The experiment was repeated 3 times.

### Transwell experiment

The 100 µL matrix glue was added to each Transwell chamber for coagulation. The cells were suspended and adjusted to 1×10^4^ cells/mL. 100μL cells were taken and added to the upper chamber of the chamber for 48 h. The cells were removed and fixed with 4% formaldehyde for 20min. The cells were stained with 0.02% crystal violet solution for 10 min Three fields of view were randomly selected to take photos.

### Scratch experiment

Cells were added into the 6-well plate at the rate of 1×10^6^ cells per well, and cultured until the fusion rate reached 100%. Samples were taken and patted at 0h, 24 h and 48h, respectively, and the scratch healing rate was assessed. The experiment was repeated three times.

### Animal studies

The experimental subjects consisted of nude mice (Hunan Slack Jingda Experimental Animal Co., LTD., Hunan, China) aged between 6 and 8 weeks. All animal procedures adhered to the guidelines for experimental animal care and use set forth by Hunan Cancer Hospital and received approval from the Ethics Committee of Hunan Cancer Hospital. Cells were digested and adjusted to concentration 1×10^7^/mL. Each nude mouse received an inoculation of 100 µL of cell lines under the right armpit skin. Nude mouse in IRF1-OE + IL2 and IRF1-KD + IL2 groups were injected with recombinant IL-2 (1 × 10^5^ units; Beijing Sihuan Biopharmaceutical Co., LTD., Beijing, China) once every 2 days, while the remaining groups received equivalent volumes of saline. The tumor-bearing mice were killed by cervical dislocation 4 weeks after the inoculation, and the tumors were removed. Tumor volume was calculated (tumor volume = (π/6)×length×width^2^).

### Single cell analysis of NSCLC

The distribution and expression of IRF1 in a single-cell dataset of NSCLC were analyzed using TISCH2 (http://tisch.comp-genomics.org/) (Sun et al., 2021). The pan-cancer risk value (HR) of this gene was also determined.

### Statistical analyses

The information can be seen as the average ± standard deviation. The software known as SPSS was used to do statistical analysis using the *t*-test or chi-square test. We evaluated the association between IRF1 expression and clinical variables using univariate analysis. The chi-squared test was used to determine the differences between the groups with high and low IRF1 levels. The medium TNFα, interferon γ, IL-2 and IL-6 and percentage of CD3, CD4, CD8, NK, B cells were identified as optimal cut-off point. The Kaplan-Meier method was applied to estimate the median PFS. Spearman’s correlation coefficient was used to calculate the relationship. *p* < 0.05 was considered to indicate a statistically significant difference.

## Results

### Different frequency changes of IRF1 and its relationship with immune regulatory factors, TMB, and MSI

**FIGURE 1:**
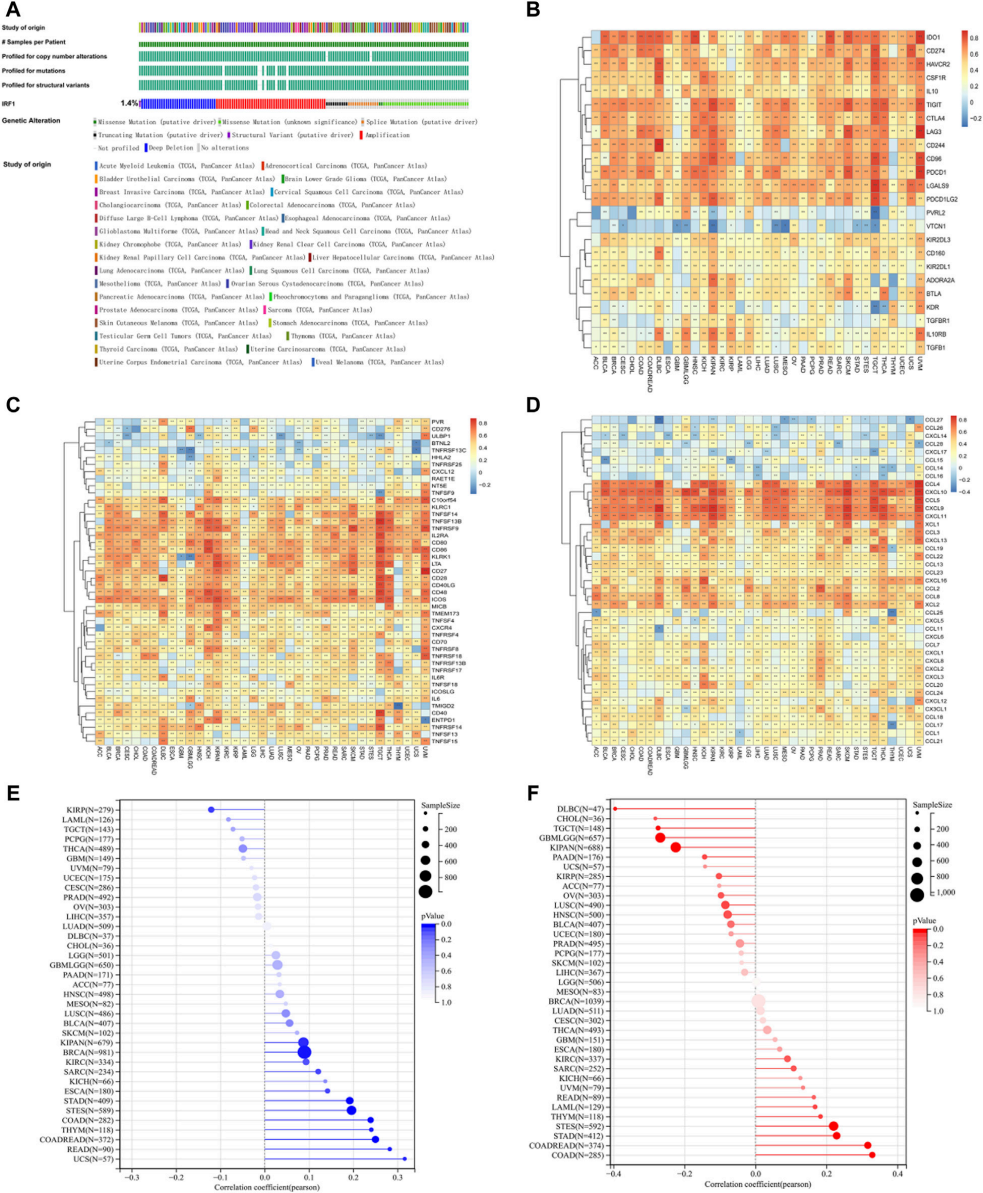
The effects of IRF1 gene in pan-cancers. **(A)**: IRF1 mutation in pan-cancer samples; The various colors in the first row of the bar graph represent various types of cancer, while the other bar graphs below represent various types of mutations. **(B)**: The correlation between IRF1 and immunosuppressive gene set in pan-cancer. The horizontal axis represents various types of cancer, the vertical axis represents the set of immunosuppressive genes, color represents the correlation coefficient, red represents a positive correlation, blue represents a negative correlation, and the darker the color represents the greater correlation; **(C)**: The correlation between IRF1 and immune stimulating gene set in pan-cancer; The horizontal axis represents various types of cancer, the vertical axis represents the set of Immune Stimulation, color represents the correlation coefficient, red represents a positive correlation, blue represents a negative correlation, and the darker the color represents the greater correlation; **(D)**: The correlation between IRF1 and chemokine gene set in pan-cancer; The horizontal axis represents various types of cancer, the vertical axis represents the set of chemokine, color represents the correlation coefficient, red represents a positive correlation, blue represents a negative correlation, and the darker the color represents the greater correlation; **(E)**: Correlation between IRF1 and TMB; **(F)**: Correlation graph between IRF1 and MSI; The horizontal axis represents the correlation coefficient, the vertical axis represents the set of various cancer genes, and the color represents the *p*-value, the darker color represents the more significant correlation.

As described before (Zhang et al., 2022), IRF1 expression was downregulated in LUAD and LUSC. According to Figure 1A, the mutation frequency of IRF1 was 1.4%, and the main mutations was deep deletions. In the immunosuppressive gene set, IRF1 positively correlated with IDO, TIGIT and LAG3 (Figure 1B). In the immune stimulation gene set, IRF1 positively correlated with IL2RA and CD80 (Figure 1C). Regarding chemokines, IRF1 positively correlated with CXCL10, CCL4 and CXCL9 (Figure 1D). IRF1 levels was closely correlated with TMB (Figure 1E) and MSI (Figure 1F). Thus, IRF1 might be closely regulate the response to immunotherapy.

### IRF1 targeted genes and functional roles

**FIGURE 2:**
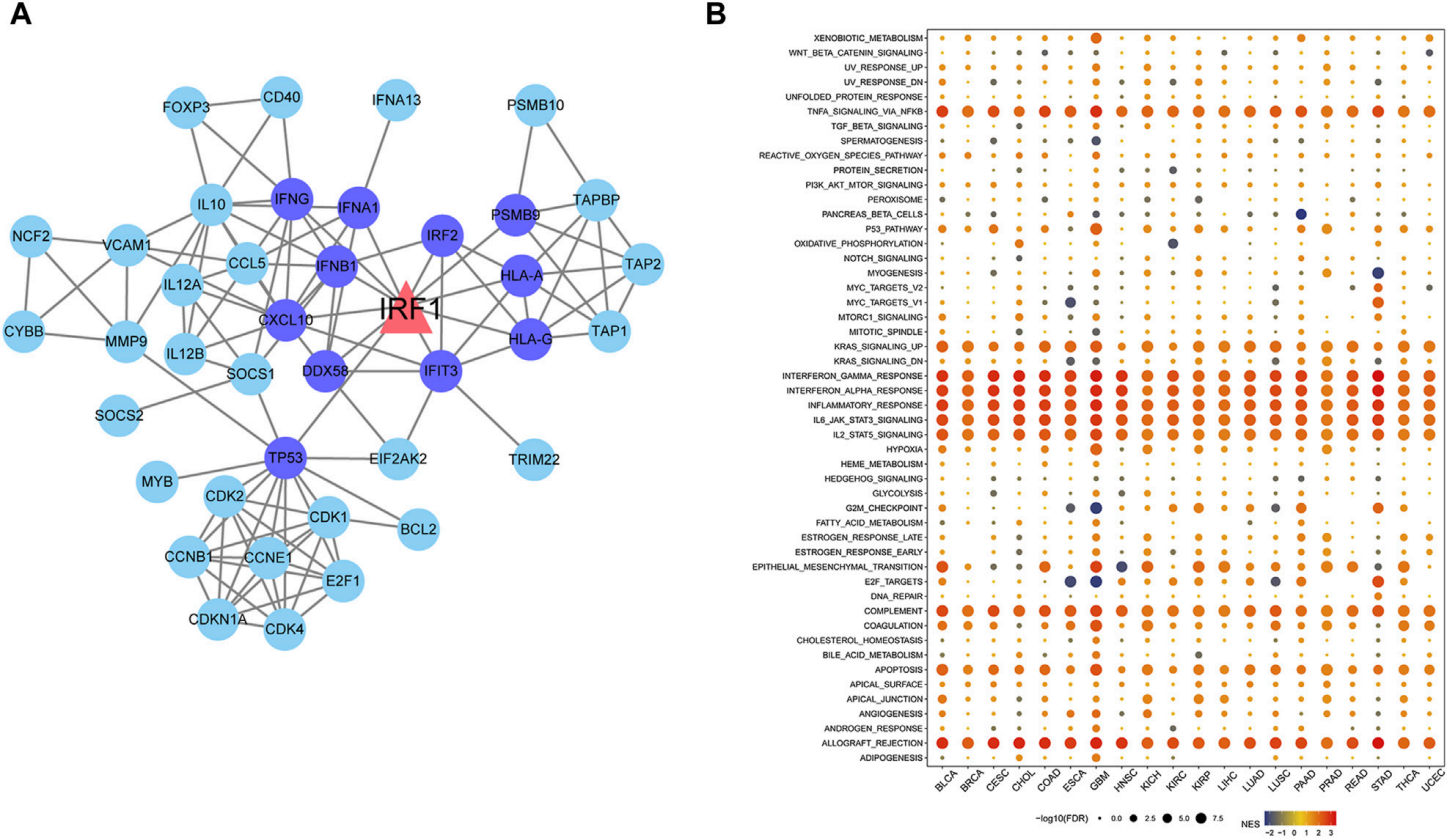
Network diagram of IRF1 target genes and pathways enriched by IRF1 based on GSEA. **(A)**: The triangle in the figure represents the target gene of IRF1, the circles are all target genes of IRF1, the dark blue represents the target gene directly related to IRF1. **(B)**: The horizontal axis represents various types of cancer, the vertical axis represents various pathways, the size of the point represents significance, the larger the point represents the more significant, and the color represents the standardized enrichment score.

A total of 57 IRF1-related genes were explored (Supplementary Table S1). TF-target gene network analysis showed that 11 genes were directly related to IRF1, including TP53, IRF2, IFNG, IFNA1, IFNB1, CXCL10 and HLA, which were closely related with interferon release and immunity regulation (Figure 2A). Differences in functional pathways were evaluated using GSEA. The pathways mainly affected by IRF1 included interferon responses and IL-2 signaling, which were mainly associated with inflammatory responses (Figure 2B). Both targeted genes and functional role analyze indicated IRF1 might play a crucial role in inflammatory responses and immunity regulation.

### Correlation analysis between IRF1 level and efficacy of chemoimmunotherapy

**FIGURE 3:**
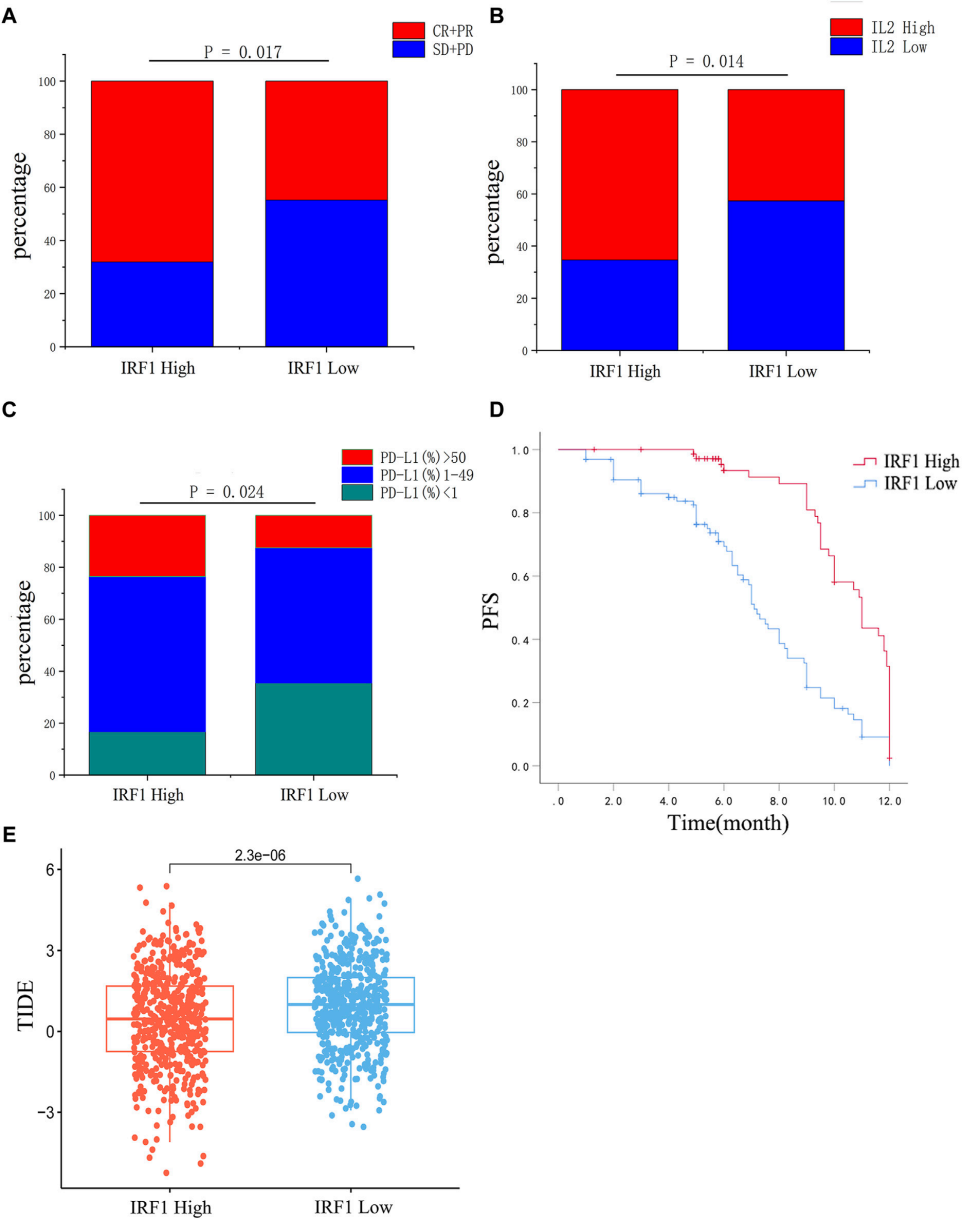
Correlation analysis between IRF1 expression and chemoimmunotherapy in NSCLC. **(A)** The efficacy of chemoimmunotherapy between the high IRF1 group and the low IRF1 group. **(B)** Expression of IL2 between high IRF1 group and low IRF1 group. **(C)** The expression of PD-L1 between the high IRF1 group and the low IRF1 group. **(D)** The expression of IRF1 is associated with PFS of first-line chemoimmunotherapy in NSCLC patients; **(E)** Box plot of IRF1 gene expression and tumor immune dysfunction and exclusion (TIDE) score. The horizontal axis represents the high and low groups distinguished by the IRF1 expression value, while the vertical axis represents the respective TIDE scores of the high and low groups. Each point in the graph represents a sample.

The baseline characteristics of the enrolled NSCLC patients are shown in Table 1. Significant differences were observed between patients with high IRF1 and low IRF1 levels for chemoimmunotherapy efficacy (Figure 3A). Patients with high IRF1 level were with higher ORR of 68.1%, while low IRF1 group with was lower ORR of 44.8% (*p* < 0.05) (Table 1). Patients of high IRF1 group were also with higher circulating IL-2 level (*p* < 0.05) (Table 1; Figure 3B) and PD-L1 distribution (*p* < 0.05) (Table 1; Figure 3C). Univariate analysis revealed that IRF1 levels were significantly associated with circulating IL-2 level and PD-L1 distribution (Table 2). High IRF1 level group was with longer PFS, a median PFS (mPFS) of 9.5 months. At 6 months, the PFS rate is 85% while at 12 months, the PFS rate is 39%. Conversely, low IRF1 group was with shorter mPFS of 5.8 months. At 6 months, the PFS rate is 48% while at 12 months, the PFS rate is 29% (Figure 3D). Clinical data supported the correlation between IRF1 level and chemoimmunotherapy efficacy and prognosis in NSCLC. Bio-informatics analyse was also used to further confirm the clinical application of IRF1 in chemoimmunotherapy. Based on the combined expression matrix of LUAD and LUSC in TCGA, patients were divided into high and low IRF1 expression groups. As shown in Figure 3E, significant differences of TIDE score were found between IRF1 high and low groups (*p* < 0.01), indicating a significant correlation between IRF1 expression immunotherapy response.

**TABLE 1:** Clinical characteristics of enrolled NSCLC patients.

Factors	IRF1 low (n = 96)	IRF1 high (n = 72)	*p*-Value
Age, n (%)			0.891
<60 years	40 (41.7)	31 (43.1)	
≥60 years	56 (58.3)	41 (56.9)	
Gender, n (%)			0.954
Male	68 (70.8)	52 (72.2)	
Female	28 (29.2)	20 (27.8)	
Pathological type, n (%)			0.623
LUSC	55 (57.3)	45 (62.5)	
LUAD	38 (39.6)	24 (33.3)	
others	3 (3.1)	3 (4.2)	
PD-L1 (%), n (%)			0.024*
<1	34 (35.4)	12 (16.7)	
1–49	50 (52.1)	43 (59.7)	
>50	12 (12.5)	17 (23.6)	
Smoking status, n (%)			0.908
≤400	42 (43.8)	30 (41.7)	
>400	54 (56.2)	42 (58.3)	
PS score, n (%)			0.637
0	8 (8.3)	5 (6.9)	
1	86 (89.6)	66 (91.7)	
2	2 (2.1)	1 (1.4)	
T stage, n (%)			0.644
1	5 (5.2)	6 (8.3)	
2	11 (11.5)	10 (13.8)	
3	48 (50.0)	34 (47.2)	
4	32 (33.3)	28 (38.9)	
N stage, n (%)			0.081
0	4 (4.2)	1 (1.4)	
1	22 (22.9)	10 (13.9)	
2	38 (39.6)	33 (45.8)	
3	32 (33.3)	28 (38.9)	
M stage, n (%)			0.389
0	10 (10.4)	9 (12.5)	
1	86 (89.6)	63 (87.5)	
TNM stage, n (%)			0.389
IIIB/IIIC	10 (10.4)	9 (12.5)	
IV	86 (89.6)	63 (87.5)	
Metastasis site, n (%)			0.187
≤3	43 (44.8)	35 (48.6)	
>3	53 (55.2)	37 (51.4)	
IL-2 median (pg/mL), n (%)			0.014*
≤7.15	55 (57.3)	25 (34.7)	
>7.15	41 (42.7)	47 (65.3)	
IL-6 median (pg/mL), n (%)			0.131
≤62.59	54 (56.3)	32 (44.4)	
>62.59	42 (43.8)	40 (55.6)	
TNFα median (pg/mL), n (%)			0.212
≤74.01	52 (54.2)	32 (44.4)	
>74.01	44 (45.8)	40 (55.6)	
Interferon γ median (ng/mL), n (%)			0.533
≤0.18	50 (52.1)	34 (47.2)	
>0.18	46 (47.9)	38 (52.8)	
CD3 cell percentage, n (%)			0.891
≤66.81	37 (38.5)	27 (37.5)	
>66.81	59 (61.5)	45 (62.5)	
CD4 cell percentage, n (%)			0.727
≤35.66	18 (18.8)	12 (16.7)	
>35.66	78 (81.3)	60 (83.3)	
CD8 cell percentage, n (%)			0.588
≤23.73	29 (30.2)	19 (26.4)	
>23.73	67 (69.8)	53 (73.6)	
B cell percentage, n (%)			0.527
≤8.43	58 (60.4)	40 (55.6)	
>8.43	38 (39.6)	32 (44.4)	
NK cell percentage, n (%)			0.752
≤21.52	13 (13.5)	11 (15.3)	
>21.52	83 (86.5)	61 (84.7)	
Chemoimmunotherapy Efficacy, n (%)			0.017*
CR + PR	43 (44.8)	49 (68.1)	
SD + PD	53 (55.2)	23 (31.9)	

* *p*-value <0.05 was considered as a significant difference.

**TABLE 2:** Cox regression analysis of significant correlation factors of IRF1.

Variables	Univariate analysis		*p*-Value
	HR	Or (95%CI)	
Age	1.003	0.896-1.122	0.959
Gender	1.616	0.109-3.524	0.585
Pathological type	4.381	0.663-2.928	0.125
PD-L1 level	0.752	0.593-1.101	0.038*
Smoke Status	2.002	0.999-2.001	0.906
PS score	1.688	0.291-9.799	0.561
T stage	0.891	0.407-1.952	0.773
N stage	2.079	0.856-5.051	0.106
M stage	1.145	0.042-3.921	0.936
TNM Stage	1.145	0.042-3.921	0.936
Metastasis site	1.594	0.798-3.167	0.187
IL-2	0.437	0.227-0.843	0.024*
IL-6	0.716	0.330-1.553	0.161
TNFα	0.869	0.417-1.809	0.707
Interferon γ	0.759	0.395-1.460	0.409
Chemoimmunotherapy Efficacy	0.824	0.682-1.214	0.041*
CD3 cell percentage	1.001	0.433-2.312	0.998
CD4 cell percentage	1.247	0.466-3.340	0.663
CD8 cell percentage	1.399	0.615-3.182	0.423
B cell percentage	1.375	0.714-2.647	0.345
NK cell percentage	0.638	0.206-1.975	0.436

* *p*-value <0.05 was considered as a significant difference.

### The synergistic effect of IRF1 and IL-2 in A549 lung cancer cells

**FIGURE 4:**
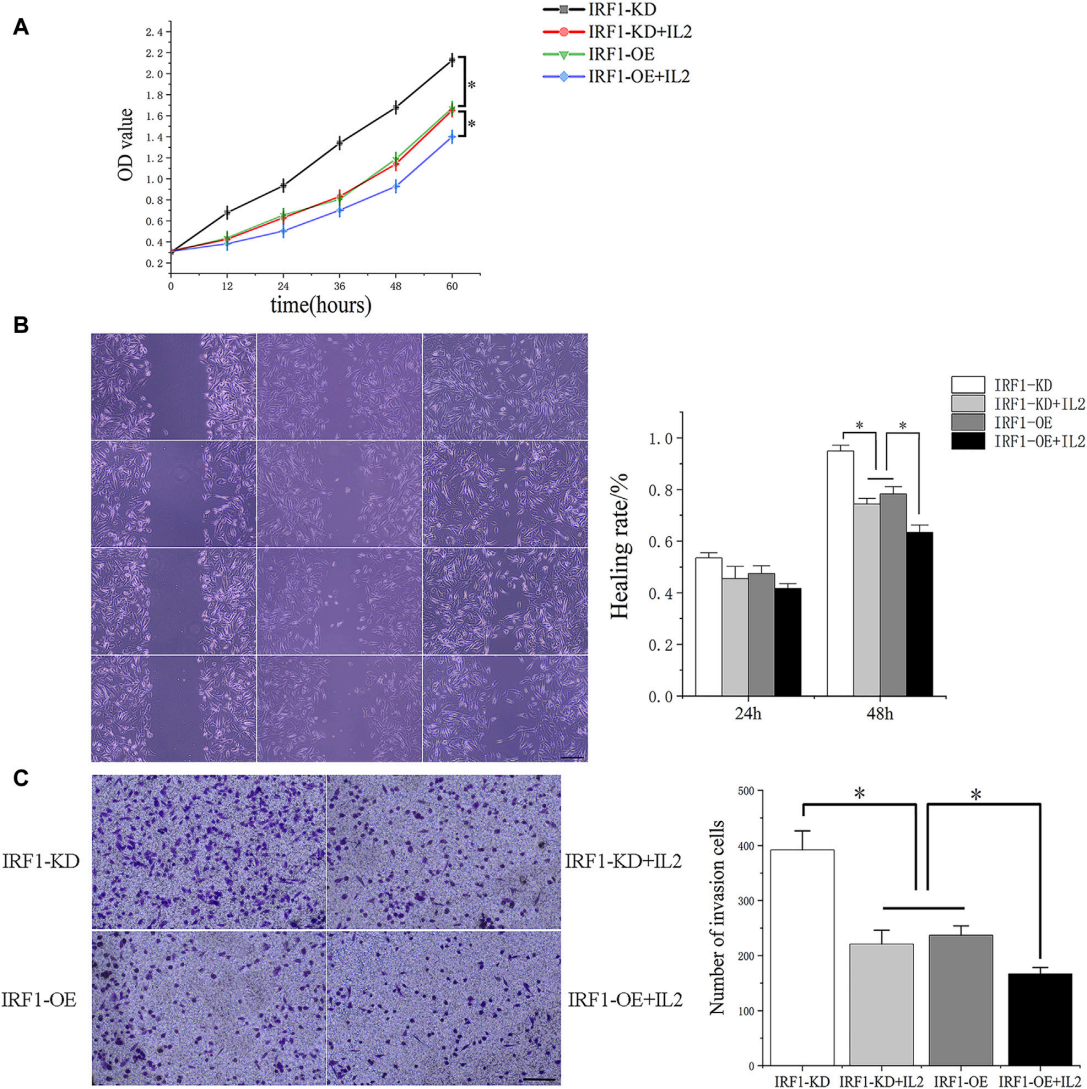
The synergistic effect of IRF1 and IL-2 in A549 lung cancer cells. **(A)** Proliferation curves of each experimental group; **(B)** The effects of IRF1 and IL2 on the healing rate of A549 cells were detected by scratch test (The scale is 100 μm); **(C)** The effects of IRF1 and IL2 on the invasion ability of A549 were detected by Transwell methodt (The scale is 100 μm). (*p < 0.05).

As showed the highest levels of IRF1 transcription and expression following cisplatin treatment (Zhang et al., 2022), A549 lung cancer cells were chosen for further *in vitro* study. IRF1 overexpression and knockdown by lentiviral and shRNA were confirmed by Western blotting and flowcytometry (data not shown). The overexpression of IRF1 significantly inhibit the proliferation, invasion and migration of A549 lung cancer cells (Figures 4A–C); and IL-2 augment the function of IRF1 on A549 lung cancer cells (Figures 4A–C). While in IRF1 knockdown group, IL-2 pretreatment mimics the effects of IRF1, including inhibition of proliferation, invasion and migration (Figures 4A–C). It is suggested that the synergistic effect of IRF1 and IL-2 in A549 lung cancer cells *in vitro*.

### The synergistic effect of IRF1 and IL-2 *in vivo*

**FIGURE 5:**
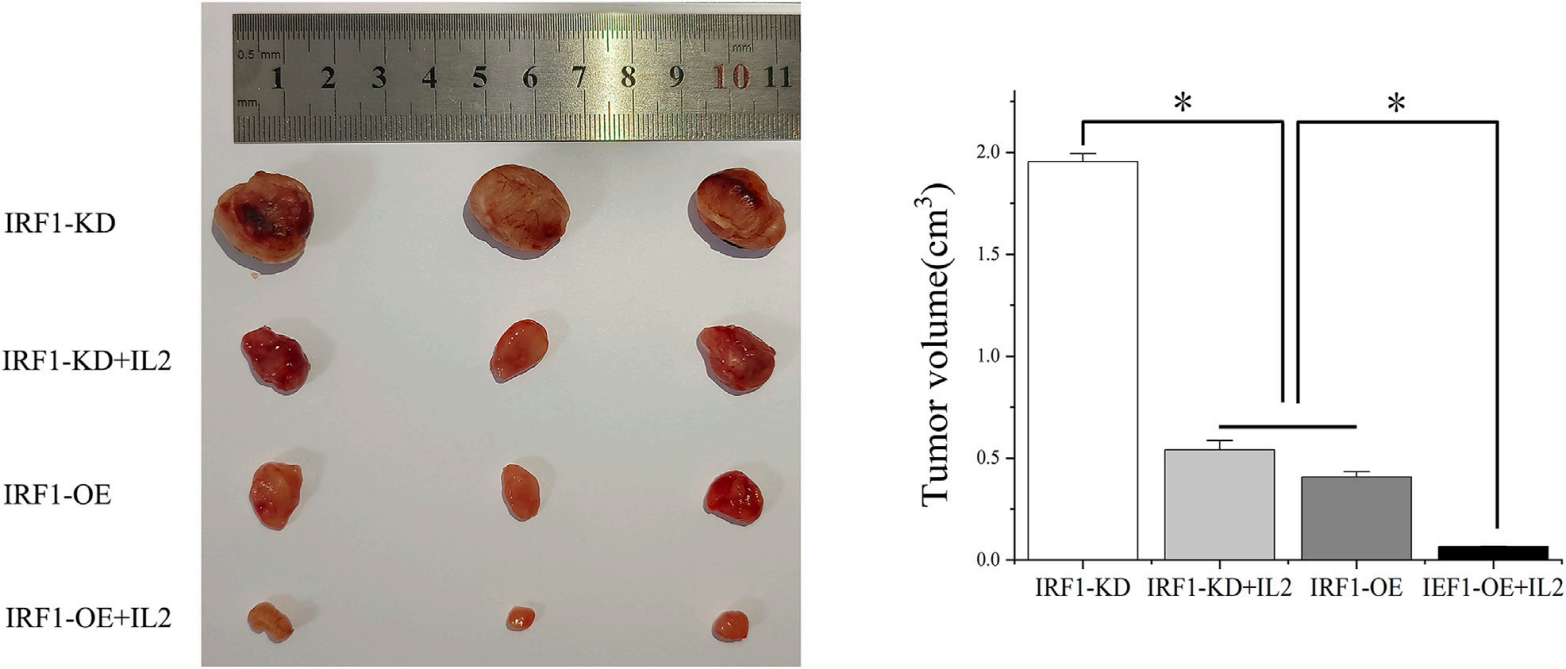
The synergistic effect of IRF1 and IL-2 *in vivo*. The synergistic effect of IRF1 and IL-2 on tumor inhibition *in vivo* was determined by subcutaneous tumor formation in nude mice. (*p < 0.05).

To further investigate the synergistic effect of IRF1 and IL-2, we transplanted IRF1 OE and IRF1 KD A549 cell line into nude mice. After 4 weeks of inoculation, we found that overexpression of IRF1 significantly inhibits the tumor growth of A549 lung cancer cells. And IL-2 injection enhanced the function of IRF1 on A549 lung cancer cells. In the IRF1 knockdown group, IL-2 treatment simulated the effect of IRF1 and significantly inhibited the tumor growth. It shows the synergistic effect of IRF1 and IL-2 *in vivo* (Figure 5).

### Single cell analysis of IRF1 in NSCLC

**FIGURE 6:**
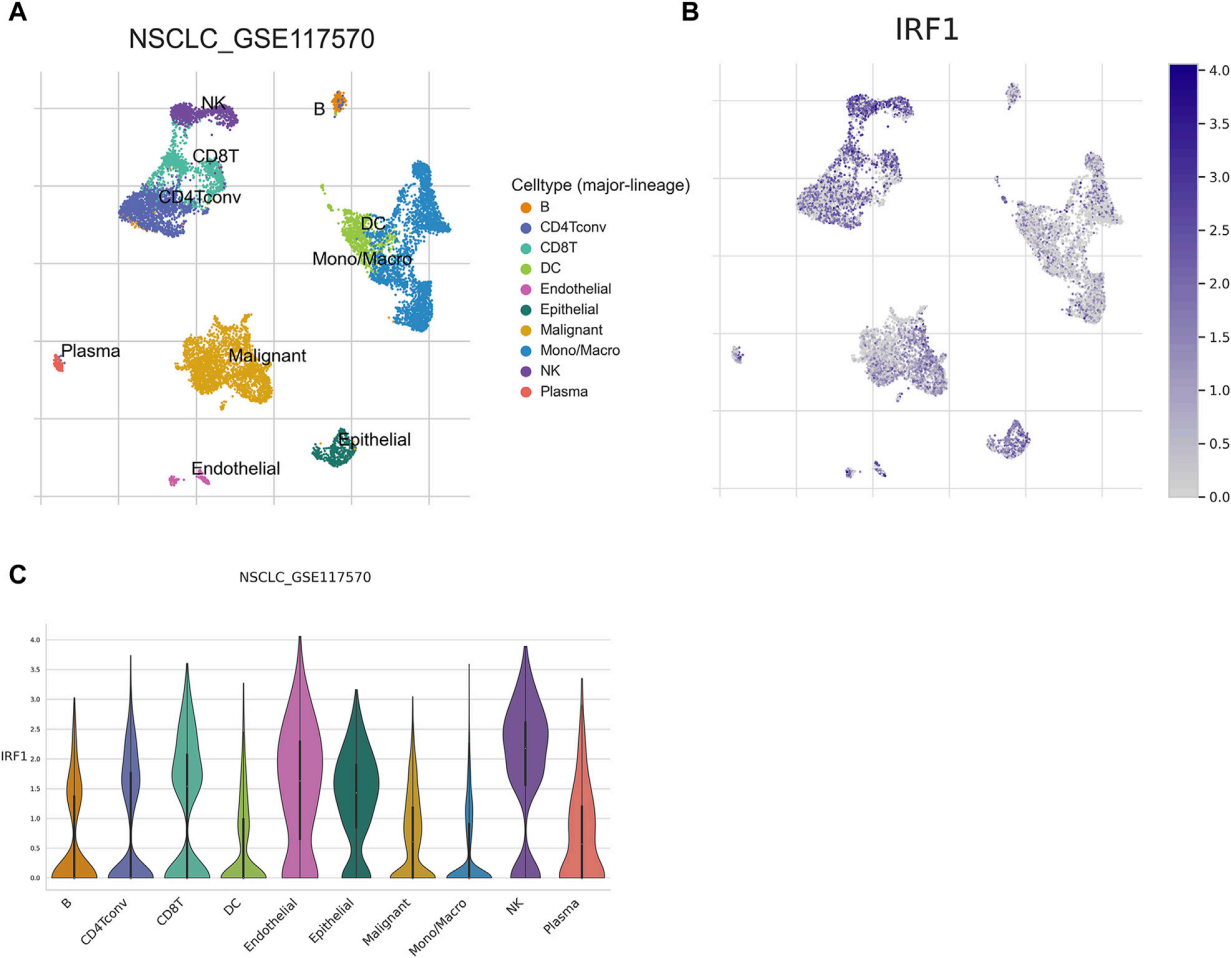
Single cell analysis of IRF1. **(A)** Single cell UMAP map; Each color represents a cell type, and each dot represents a cell. **(B)** Scatter plot of IRF1 single cell expression distribution; the darker the color represents the higher expression, and each dot represents a cell; **(C)** Violin diagram of IRF1 expression in cells; The horizontal axis represents the cell type, while the vertical axis represents the expression level of IRF1 in each cell type.

Given the *in vitro* synergistic anti-tumor function of IRF1 and IL-2, we further explore the potential immune cell types which might play a major role in the complex tumor microenvironment. The expression of IRF1 in NSCLC single-cell data was analyzed using the TISCH2 database, and GSE117570 was used to perform IRF1-based single-cell analysis. UMAP revealed 11 cell sub-types, including NK, endothelial, and malignant cells (Figure 6A). The expression levels of IRF1 in each cell types were further analyzed. The expression level of IRF1 was higher in NK and endothelial cells than other sub-types (Figures 6B, C). Further study should be focused on NK cells and related receptor to explore the interplay between IRF1 and IL-2 *in vivo*.

## Discussion

**FIGURE 7:**
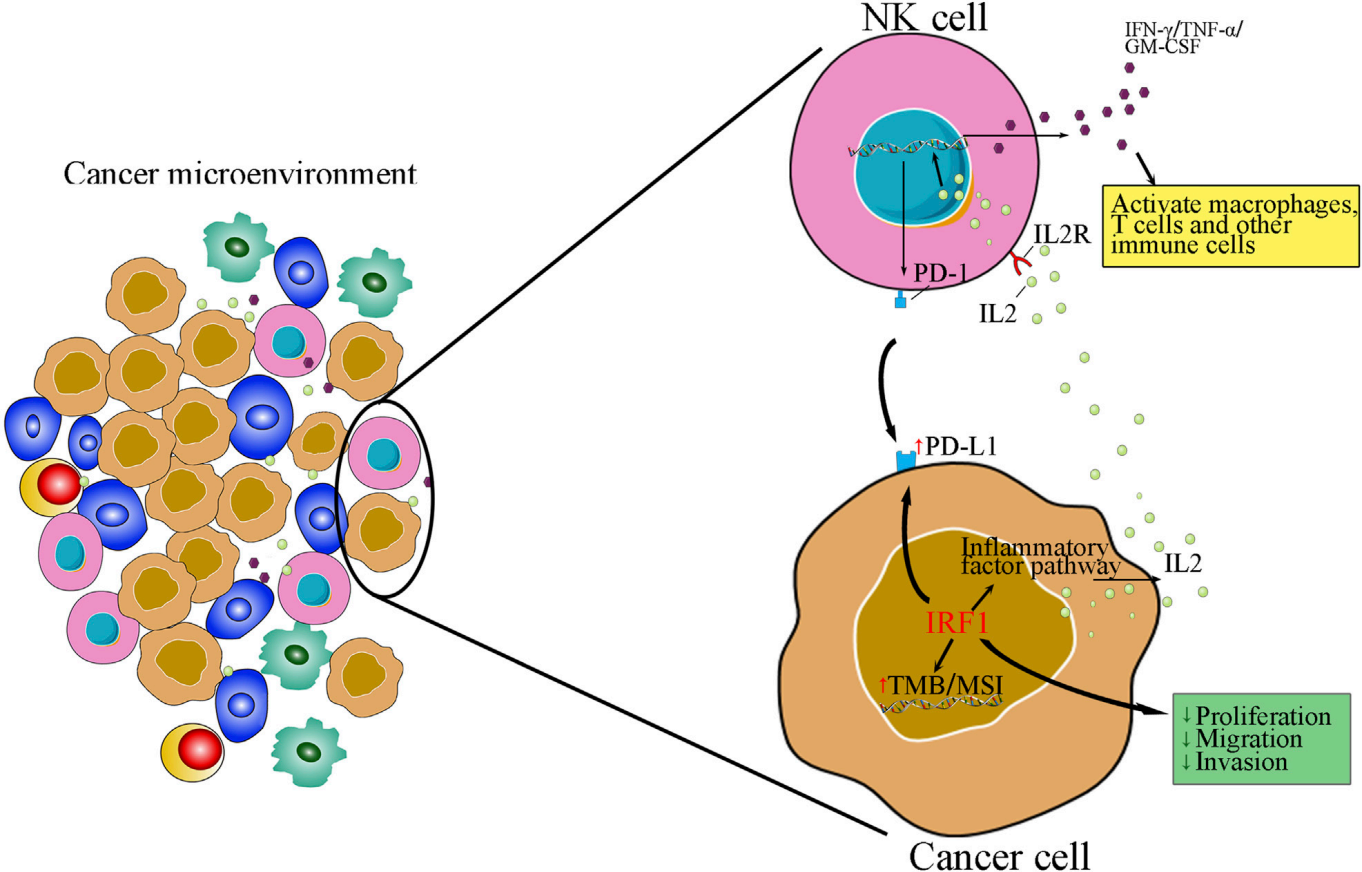
An schematic of how interplay of IRF1 and IL-2 in tumour micro-environment in NSCLC.

The critical function of IRF1 in the immune systems of various cancers has been explored (Kirchhoff et al., 1993; Kim et al., 2002), the clinical application and biological function of IRF1 in NSCLC patients who receiving chemoimmunotherapy remains unknown. In our investigation, we discovered using bioinformatics analysis that IRF1 is intimately associated with the control of immunological activation, potentially impacting the outcome of immunotherapy. IRF1 also had a role in the control of other inflammatory responses, such as IL2 signaling. Clinical research has revealed a significant correlation between IRF1 and the level of circulating IL-2, which is linked to the effectiveness and prognosis of chemotherapy for non-small cell lung cancer. IRF1 and IL2 have a synergistic impact that inhibits the proliferation, migration, and invasion of A549 lung cancer cells, as demonstrated by both *in vitro* and *in vivo* tests (Figure 7).

Our data showed that IRF1 expression levels were correlated with immunosuppressive genes, such as IDO, TIGIT and LAG3, and immune stimulation genes, such as IL2RA and CD80. Meanwhile, the gene was also closely correlated with TMB and MSI. Extensive researches have confirmed that, the factors, including immunosuppressive and immune-stimulatory gene sets, TMB, and MSI, were all valuable predictive biomarkers for immunotherapy (Chang et al., 2018; Strickler et al., 2021; Turan et al., 2021). Therefore, IRF1 might be a critical biomarker for evaluating treatment efficacy for patients receiving immunotherapy. Furthermore, we have also observed that high IRF1 level group was with longer PFS, and low IRF1 group was with shorter PFS. Meanwhile, clinical data also supported the correlation between IRF1 level and prognosis in NSCLC. Therefore, patients with higher IRF1 levels might be more easily to achieve benefit from immunotherapy in clinic. Interestingly, IRF1-mediated PDL1 levels have been reported previously (Sato et al., 2017). Moreover, tumor immune microenvironment plays a role in interferon γ stimulated PD-L1 expression in tumors (Bally et al., 2016; Liu et al., 2018). In circulating tumor cells, PD-L1 and IRF1 expression levels are all associated with immunotherapy efficacy (Kennedy et al., 2019). Therefore, IRF1, along with PD-L1, might serve as a predictive biomarker for immunotherapy response.

Cancer-associated inflammation affects malignancy related events, including tumor metastasis, angiogenesis, survival, and proliferation (Gomes et al., 2014). We showed that IRF1 is mainly involved in pathways associated with inflammation and interferon, such as interferon γ and IL-2 signaling. The critical role of the IL-2 signaling pathway in various cancers has been extensively studied (Alvarez et al., 2006). IRF1 and IL-2 are both involved in immune response while IRF1 may influence the expression of genes involved in immune responses, including those related to IL-2 (Konjevic et al., 2010; Perazzio et al., 2017). Utilizing IL-2 successfully counteracts the inhibitory impact of elevated IRF1 expression on the proliferation, migration, and invasion of lung adenocarcinoma cells. It is worth noting that the specific regulation between IRF1 and IL-2 may vary depending on the context of immune response. The intricate interplay between various transcription factors and cytokines ensures a coordinated and effective immune response (Devenish et al., 2021). In our study, the synergistic effect of IRF1 and IL2 has been found in chemotherapy combined with immunotherapy in NSCLC. Therefore, these results indicate that both IRF1 and IL2 are key components of antitumor immunity.

We also found that IRF1 levels were higher in NK cells, suggesting that IRF1 might mediate NK cell induced immunity regulation. Previously, Gungabeesoonv et al. revealed that loss of IRF1 in neutrophils would lead to failure of immunotherapy (Gungabeesoon et al., 2023). Meanwhile, neutrophils have been shown to suppress the NK cell infiltration, by downregulating CCR1 and to impair anti-tumor capabilities by cell-to-cell interactions, through the PD-L1/PD-1 axis (Sun et al., 2020). IRF1 was previously demonstrated to be tumor suppressor gene mediated by increasing the secretion of activated NK cells migration (Yan et al., 2021). NK cells are valuable in generating an antitumor effect, and immunotherapy targeting NK cells are recognized as promising therapeutic strategies for treating tumors (Wang et al., 2022). Additionally, IL2 might enhance NK cytotoxicity (Hernandez et al., 2022). Single cell sequencing suggests that NK cells with IRF1 overexpression in NSCLC (Figure 7). Thus, targeting IRF1/IL2 axis may be used to modulate immunotherapy-elicited NK cells and neutrophil responses via cell-to-cell interactions. However, the function of NK cells and detailed mechanisms of correlation between IRF1 and IL2 in immunotherapy responses requires further validation by observing NK cell proliferation, invasion and migration mediated by IRF1 and IL2 levels. Meanwhile, the secretion of immune related cytokines, such as IFN-γ, TNF-β, should also be recorded. Furthermore, our previous data have shown that IRF-1 levels could regulate mitochondrial depolarization, oxidative stress, and autophagy in A549 cells (Zhang et al., 2022). These processes might also occur among subjects underwent chemoimmunotherapy. Therefore, further *in vitro* and *in vivo* study should be designed to observe cellular processes such as autophagy, apoptosis, and mitochondrial homeostasis in cell models with overexpression or downregulation IRF1 via flow cytometry and corresponding assay kits.

In summary, IRF1 might be with predictive and prognostic value for chemoimmunotherapy in NSCLC patients through regulation of inflammatory pathway, including IL-2. However, further research is needed to explore the underlying mechanisms.

## Data Availability

The original contributions presented in the study are included in the article/Supplementary Material, further inquiries can be directed to the corresponding author.

## Supplementary material

SUPPLEMENTARY FIGURE S1 Representative images of IRF1 immunohistochemical staining in non-small-cell lung carcinomas. A High IRF1 expression in squamous cell carcinoma; B low IRF1 expression in squamous cell carcinoma; C High IRF1 expression in adenocarcinoma; D low IRF1 expression in adenocarcinoma.

SUPPLEMENTARY TABLE S1 Clinical characteristics of enrolled NSCLC patients.
