# Supplementary material for: Clinical significance and biological function of interferon regulatory factor 1 in non-small cell lung cancer
Source: Front Pharmacol. 2024 Jun 10;15:1413699. doi: 10.3389/fphar.2024.1413699 (PMC11194705; doi:10.3389/fphar.2024.1413699)
Supplement: Supplementary file 1 [file Table1.docx]

Supplementary Table 1 The predicted genes targeted by IRF1

| TF | Target | Mode of Regulation | References(PMID) |
| --- | --- | --- | --- |
| IRF1 | [BCL2](http://www.genecards.org/index.php?path=/Search/Symbol/BCL2) | Repression | [10918594](http://www.ncbi.nlm.nih.gov/pubmed/?term=10918594) |
| IRF1 | [CASP7](http://www.genecards.org/index.php?path=/Search/Symbol/CASP7) | Activation | [10918594](http://www.ncbi.nlm.nih.gov/pubmed/?term=10918594) |
| IRF1 | [CCL5](http://www.genecards.org/index.php?path=/Search/Symbol/CCL5) | Unknown | [10385645](http://www.ncbi.nlm.nih.gov/pubmed/?term=10385645) |
| IRF1 | [CCNB1](http://www.genecards.org/index.php?path=/Search/Symbol/CCNB1) | Repression | [22200613](http://www.ncbi.nlm.nih.gov/pubmed/?term=22200613) |
| IRF1 | [CCNE1](http://www.genecards.org/index.php?path=/Search/Symbol/CCNE1) | Repression | [22200613](http://www.ncbi.nlm.nih.gov/pubmed/?term=22200613) |
| IRF1 | [CD40](http://www.genecards.org/index.php?path=/Search/Symbol/CD40) | Activation | [18694960](http://www.ncbi.nlm.nih.gov/pubmed/?term=18694960) |
| IRF1 | [CDK1](http://www.genecards.org/index.php?path=/Search/Symbol/CDK1) | Repression | [22200613](http://www.ncbi.nlm.nih.gov/pubmed/?term=22200613) |
| IRF1 | [CDK2](http://www.genecards.org/index.php?path=/Search/Symbol/CDK2) | Repression | [22200613](http://www.ncbi.nlm.nih.gov/pubmed/?term=22200613) |
| IRF1 | [CDK4](http://www.genecards.org/index.php?path=/Search/Symbol/CDK4) | Repression | [22200613](http://www.ncbi.nlm.nih.gov/pubmed/?term=22200613) |
| IRF1 | [CDKN1A](http://www.genecards.org/index.php?path=/Search/Symbol/CDKN1A) | Repression | [12531694](http://www.ncbi.nlm.nih.gov/pubmed/?term=12531694) |
| IRF1 | [CDKN1A](http://www.genecards.org/index.php?path=/Search/Symbol/CDKN1A) | Unknown | 12420214; 15509808 |
| IRF1 | [CEACAM1](http://www.genecards.org/index.php?path=/Search/Symbol/CEACAM1) | Activation | [21050451](http://www.ncbi.nlm.nih.gov/pubmed/?term=21050451) |
| IRF1 | [CIITA](http://www.genecards.org/index.php?path=/Search/Symbol/CIITA) | Activation | 10557076; 11464288 |
| IRF1 | [CIITA](http://www.genecards.org/index.php?path=/Search/Symbol/CIITA) | Unknown | 10202014; 9916712 |
| IRF1 | [CXCL10](http://www.genecards.org/index.php?path=/Search/Symbol/CXCL10) | Activation | [19342664](http://www.ncbi.nlm.nih.gov/pubmed/?term=19342664) |
| IRF1 | [CYBB](http://www.genecards.org/index.php?path=/Search/Symbol/CYBB) | Activation | [9593745](http://www.ncbi.nlm.nih.gov/pubmed/?term=9593745) |
| IRF1 | [DDX58](http://www.genecards.org/index.php?path=/Search/Symbol/DDX58) | Activation | [22391244](http://www.ncbi.nlm.nih.gov/pubmed/?term=22391244) |
| IRF1 | [DST](http://www.genecards.org/index.php?path=/Search/Symbol/DST) | Repression | [15560761](http://www.ncbi.nlm.nih.gov/pubmed/?term=15560761) |
| IRF1 | [E2F1](http://www.genecards.org/index.php?path=/Search/Symbol/E2F1) | Repression | [22200613](http://www.ncbi.nlm.nih.gov/pubmed/?term=22200613) |
| IRF1 | [EIF2AK2](http://www.genecards.org/index.php?path=/Search/Symbol/EIF2AK2) | Activation | [8622878](http://www.ncbi.nlm.nih.gov/pubmed/?term=8622878) |
| IRF1 | [ERAP2](http://www.genecards.org/index.php?path=/Search/Symbol/ERAP2) | Unknown | [15691326](http://www.ncbi.nlm.nih.gov/pubmed/?term=15691326) |
| IRF1 | [FASLG](http://www.genecards.org/index.php?path=/Search/Symbol/FASLG) | Unknown | [14563113](http://www.ncbi.nlm.nih.gov/pubmed/?term=14563113) |
| IRF1 | [FOXP3](http://www.genecards.org/index.php?path=/Search/Symbol/FOXP3) | Repression | [18641303](http://www.ncbi.nlm.nih.gov/pubmed/?term=18641303) |
| IRF1 | [HLA-A](http://www.genecards.org/index.php?path=/Search/Symbol/HLA-A) | Activation | [19428110](http://www.ncbi.nlm.nih.gov/pubmed/?term=19428110) |
| IRF1 | [HLA-G](http://www.genecards.org/index.php?path=/Search/Symbol/HLA-G) | Unknown | [11087747](http://www.ncbi.nlm.nih.gov/pubmed/?term=11087747) |
| IRF1 | [IFIT3](http://www.genecards.org/index.php?path=/Search/Symbol/IFIT3) | Activation | [21056555](http://www.ncbi.nlm.nih.gov/pubmed/?term=21056555) |
| IRF1 | [IFNA1](http://www.genecards.org/index.php?path=/Search/Symbol/IFNA1) | Activation | [12077266](http://www.ncbi.nlm.nih.gov/pubmed/?term=12077266) |
| IRF1 | [IFNA1](http://www.genecards.org/index.php?path=/Search/Symbol/IFNA1) | Unknown | [7606736](http://www.ncbi.nlm.nih.gov/pubmed/?term=7606736) |
| IRF1 | [IFNA13](http://www.genecards.org/index.php?path=/Search/Symbol/IFNA13) | Activation | [12077266](http://www.ncbi.nlm.nih.gov/pubmed/?term=12077266) |
| IRF1 | [IFNB1](http://www.genecards.org/index.php?path=/Search/Symbol/IFNB1) | Activation | [8021262](http://www.ncbi.nlm.nih.gov/pubmed/?term=8021262) |
| IRF1 | [IFNB1](http://www.genecards.org/index.php?path=/Search/Symbol/IFNB1) | Unknown | 17073600; 18575764; 22547704 |
| IRF1 | [IFNG](http://www.genecards.org/index.php?path=/Search/Symbol/IFNG) | Activation | [13678429](http://www.ncbi.nlm.nih.gov/pubmed/?term=13678429) |
| IRF1 | [IL10](http://www.genecards.org/index.php?path=/Search/Symbol/IL10) | Activation | [12817009](http://www.ncbi.nlm.nih.gov/pubmed/?term=12817009) |
| IRF1 | [IL12A](http://www.genecards.org/index.php?path=/Search/Symbol/IL12A) | Unknown | [16622221](http://www.ncbi.nlm.nih.gov/pubmed/?term=16622221) |
| IRF1 | [IL12B](http://www.genecards.org/index.php?path=/Search/Symbol/IL12B) | Unknown | [10657616](http://www.ncbi.nlm.nih.gov/pubmed/?term=10657616) |
| IRF1 | [IL27](http://www.genecards.org/index.php?path=/Search/Symbol/IL27) | Activation | 17684041; 20083668 |
| IRF1 | [IRF2](http://www.genecards.org/index.php?path=/Search/Symbol/IRF2) | Activation | [9054665](http://www.ncbi.nlm.nih.gov/pubmed/?term=9054665) |
| IRF1 | [MMP9](http://www.genecards.org/index.php?path=/Search/Symbol/MMP9) | Unknown | [12105194](http://www.ncbi.nlm.nih.gov/pubmed/?term=12105194) |
| IRF1 | [MYB](http://www.genecards.org/index.php?path=/Search/Symbol/MYB) | Repression | [10739671](http://www.ncbi.nlm.nih.gov/pubmed/?term=10739671) |
| IRF1 | [NCF2](http://www.genecards.org/index.php?path=/Search/Symbol/NCF2) | Activation | [10570299](http://www.ncbi.nlm.nih.gov/pubmed/?term=10570299) |
| IRF1 | [OGN](http://www.genecards.org/index.php?path=/Search/Symbol/OGN) | Unknown | [12835654](http://www.ncbi.nlm.nih.gov/pubmed/?term=12835654) |
| IRF1 | [PIGR](http://www.genecards.org/index.php?path=/Search/Symbol/PIGR) | Unknown | [9973374](http://www.ncbi.nlm.nih.gov/pubmed/?term=9973374) |
| IRF1 | [POLH](http://www.genecards.org/index.php?path=/Search/Symbol/POLH) | Unknown | [22367195](http://www.ncbi.nlm.nih.gov/pubmed/?term=22367195) |
| IRF1 | [PSMB10](http://www.genecards.org/index.php?path=/Search/Symbol/PSMB10) | Unknown | [18694960](http://www.ncbi.nlm.nih.gov/pubmed/?term=18694960) |
| IRF1 | [PSMB9](http://www.genecards.org/index.php?path=/Search/Symbol/PSMB9) | Activation | [18694960](http://www.ncbi.nlm.nih.gov/pubmed/?term=18694960) |
| IRF1 | [PSMB9](http://www.genecards.org/index.php?path=/Search/Symbol/PSMB9) | Unknown | 10764778; 10921891; 16703666; 9632673 |
| IRF1 | [SLPI](http://www.genecards.org/index.php?path=/Search/Symbol/SLPI) | Unknown | [10498899](http://www.ncbi.nlm.nih.gov/pubmed/?term=10498899) |
| IRF1 | [SOCS1](http://www.genecards.org/index.php?path=/Search/Symbol/SOCS1) | Activation | [20644166](http://www.ncbi.nlm.nih.gov/pubmed/?term=20644166) |
| IRF1 | [SOCS2](http://www.genecards.org/index.php?path=/Search/Symbol/SOCS2) | Activation | [22291912](http://www.ncbi.nlm.nih.gov/pubmed/?term=22291912) |
| IRF1 | [TAP1](http://www.genecards.org/index.php?path=/Search/Symbol/TAP1) | Activation | [18694960](http://www.ncbi.nlm.nih.gov/pubmed/?term=18694960) |
| IRF1 | [TAP1](http://www.genecards.org/index.php?path=/Search/Symbol/TAP1) | Unknown | [9632673](http://www.ncbi.nlm.nih.gov/pubmed/?term=9632673) |
| IRF1 | [TAP2](http://www.genecards.org/index.php?path=/Search/Symbol/TAP2) | Unknown | [18694960](http://www.ncbi.nlm.nih.gov/pubmed/?term=18694960) |
| IRF1 | [TAPBP](http://www.genecards.org/index.php?path=/Search/Symbol/TAPBP) | Unknown | [18694960](http://www.ncbi.nlm.nih.gov/pubmed/?term=18694960) |
| IRF1 | [TNFSF10](http://www.genecards.org/index.php?path=/Search/Symbol/TNFSF10) | Activation | [21858676](http://www.ncbi.nlm.nih.gov/pubmed/?term=21858676) |
| IRF1 | [TP53](http://www.genecards.org/index.php?path=/Search/Symbol/TP53) | Unknown | [19003964](http://www.ncbi.nlm.nih.gov/pubmed/?term=19003964) |
| IRF1 | [TRIM22](http://www.genecards.org/index.php?path=/Search/Symbol/TRIM22) | Unknown | 21683060; 23670564; 23729439 |
| IRF1 | [VCAM1](http://www.genecards.org/index.php?path=/Search/Symbol/VCAM1) | Unknown | 15265939; 22182512 |
