# Supplementary figures and images for: Clinical significance and biological function of interferon regulatory factor 1 in non-small cell lung cancer
Source: Front Pharmacol. 2024 Jun 10;15:1413699. doi: 10.3389/fphar.2024.1413699 (PMC11194705; doi:10.3389/fphar.2024.1413699)

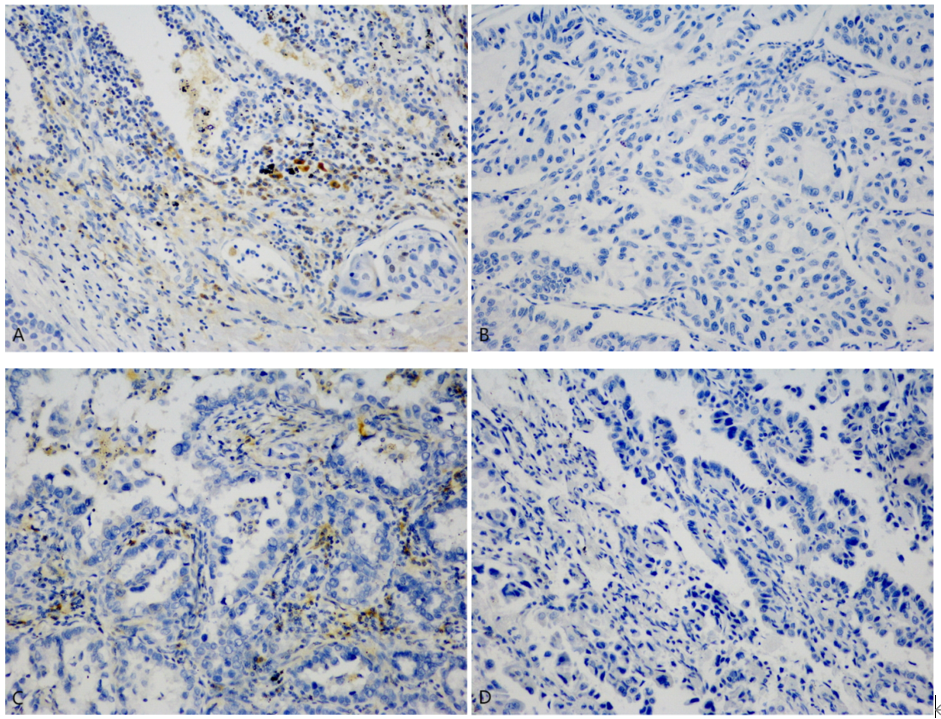

Supplement: Supplementary file 2 [file Image1.TIF]
